# Haplotype-resolved telomere-to-telomere assembly and haplotype-aware annotation pipeline enable high-quality reannotation of three *Citrus* genomes

**DOI:** 10.1093/hr/uhag048

**Published:** 2026-02-28

**Authors:** Jing Huang, Pei-Xuan Xiao, Ling Cui, Lei Tan, Shenchao Zhu, Junli Ye, Wen-Biao Jiao

**Affiliations:** National Key Laboratory for Germplasm Innovation and Utilization of Horticultural Crops, Huazhong Agricultural University, Wuhan 430070, China; Hubei Hongshan Laboratory, Wuhan 430070, China; Hubei Key Laboratory of Agricultural Bioinformatics, College of Informatics, Huazhong Agricultural University, Wuhan 430070, China; National Key Laboratory for Germplasm Innovation and Utilization of Horticultural Crops, Huazhong Agricultural University, Wuhan 430070, China; Hubei Hongshan Laboratory, Wuhan 430070, China; Hubei Key Laboratory of Agricultural Bioinformatics, College of Informatics, Huazhong Agricultural University, Wuhan 430070, China; National Key Laboratory for Germplasm Innovation and Utilization of Horticultural Crops, Huazhong Agricultural University, Wuhan 430070, China; Hubei Hongshan Laboratory, Wuhan 430070, China; Hubei Key Laboratory of Agricultural Bioinformatics, College of Informatics, Huazhong Agricultural University, Wuhan 430070, China; National Key Laboratory for Germplasm Innovation and Utilization of Horticultural Crops, Huazhong Agricultural University, Wuhan 430070, China; Hubei Hongshan Laboratory, Wuhan 430070, China; Hubei Key Laboratory of Agricultural Bioinformatics, College of Informatics, Huazhong Agricultural University, Wuhan 430070, China; National Key Laboratory for Germplasm Innovation and Utilization of Horticultural Crops, Huazhong Agricultural University, Wuhan 430070, China; National Key Laboratory for Germplasm Innovation and Utilization of Horticultural Crops, Huazhong Agricultural University, Wuhan 430070, China; National Key Laboratory for Germplasm Innovation and Utilization of Horticultural Crops, Huazhong Agricultural University, Wuhan 430070, China; Hubei Hongshan Laboratory, Wuhan 430070, China; Hubei Key Laboratory of Agricultural Bioinformatics, College of Informatics, Huazhong Agricultural University, Wuhan 430070, China

## Abstract

*Citrus* species are economically and nutritionally vital, with their fruits cultivated globally. Despite the publication of multiple genomes for *Citrus*, high-quality assemblies that achieve both haplotype resolution and telomere-to-telomere (HR-T2T) continuity remain scarce—pummelo (*Citrus maxima*) being a notable example. Compounded by limitations in gene annotation quality, these gaps hinder functional genomic research and genomics-assisted breeding. Here, we report the first high-quality HR–T2T genome assembly of pummelo, generated using PacBio HiFi and Oxford Nanopore sequencing. The two haplotype assemblies presented contig N50 values of 38.58 and 32.57 Mb, completeness scores of 99.36% and 99.66%, and nucleotide accuracies of 99.99994% and 99.99997%, respectively. We developed HapGene, a haplotype-aware annotation pipeline that integrates short-read RNA-Seq and long-read Iso-Seq data to enable unbiased annotation. Benchmarking showed HapGene captured 3% to 10% of genes missed or misannotated by conventional pipelines and reduces false haplotype-specific genes by 4- to 5-fold. Leveraging 380 Gb of newly sequenced and 2792 Gb of public transcriptomic data, we comprehensively annotated protein-coding and non-coding genes across three major *Citrus* crops (sweet orange, pummelo, and mandarin). This effort revealed 18 757–21 083 alternative splicing events, 1725–1853 resistance gene analogues, and 2392–3757 long intergenic RNAs (lincRNAs). Genomic and transcriptomic characterization of lincRNAs indicated their functional innovation (many associated with stress responses) in *Citrus*. Additionally, we revealed around one-third of genes exhibited tissue-specific allelic differential expression. Our work provides a critical genomic resource and analytical tool to advance *Citrus* genomic research, thereby driving progress in functional and evolutionary genomics while laying a robust foundation for precise genomics-assisted breeding.

## Introduction

The genus *Citrus*, belonging to the family Rutaceae, comprises economically important fruit crops worldwide, including sweet orange (*Citrus sinensis*), pummelo (*Citrus maxima*), and mandarin (*Citrus reticulata*) [1]. Cultivated in over 140 countries, these crops yield ~150 million tons annually (according to FAO statistics, 2023, https://www.fao.org/faostat/en/). *Citrus* fruits possess significant nutritional, medical, and cosmetic values [2]. However, due to their complex domestication history and extensive interspecific hybridization, the genomic architecture of this genus is highly intricate, and taxonomic relationships remain obscure [2, 3]. These challenges hinder functional gene research and cultivar improvement efforts. Therefore, obtaining high-quality, haplotype-resolved genome assemblies is essential for elucidating the evolutionary relationships and domestication history within the *Citrus* genus [4]. Moreover, haplotype-resolved and telomere-to-telomere (T2T) assembly enables comprehensive exploration of allelic sequence and expression divergence across both normal and difficult-to-access genomic regions, which further benefit to pinpoint the trait-associated allele-specific variants and improve the gene editing efficiency in heterozygous regions [5–7].

In recent years, more than 50 *Citrus* genome assemblies have been reported [8–11], varying in continuity, completeness, and haplotype resolution. A recent study released the first haplotype-resolved T2T assembly of sweet orange and sour orange [12]. However, no such assembly has been reported for pummelo, one of three basic *Citrus* species. Wang *et al*. [13] first reported a haploid draft genome of pummelo, but the scaffold-level assembly was highly fragmented (1612 scaffolds with an N50 of 4.2 Mb, 2602 contigs with an N50 of 2.2 Mb). Subsequently, Lu *et al*. [14] generated a chromosome-scale pummelo genome assembly with a contig N50 of 3.8 Mb. Zheng *et al*. [15] further assembled a chromosome-scale genome of the medicinal cultivar ‘Huazhouyou-tomentosa’ (HZY-T) with a contig N50 of 1.74 Mb. Most recently, Liu *et al*. published a gap-free T2T pummelo genome assembly for the cultivar ‘PingShan’, but haplotype information remained unresolved [16]. As one of the three ancestral species of modern *Citrus* hybrids, together with mandarin *(Citrus reticulata)* and citron *(Citrus medica)*, pummelo occupies a basal position in the *Citrus* phylogeny [13]. It serves as the maternal or paternal progenitor of many important cultivated species (e.g. sweet orange) [12]. Due to its pivotal evolutionary role, resolving the pummelo haplotype genome is essential for understanding hybrid speciation and allelic diversity in *Citrus*, yet incomplete haplotype characterization still limits such insights.

Despite these advances, current studies suffer from critical limitations in gene annotation of haplotype-resolved assemblies and pangenomes. These include incomplete protein-coding gene annotations, insufficient identification of non-coding elements such as long intergenic RNAs (lincRNAs), and misannotation of gene boundaries [17, 18]. Traditional annotation approaches, which rely on single-genome references, overlook haplotype relationships, leading to biased, inaccurate allelic annotations and substantial false haplotype-specific gene models. Additionally, RNA-Seq, the typical data for annotation, has limited read length (100–250 bp), failing to detect long splicing junctions and cover full transcripts. In contrast, long and accurate transcriptome sequencing reads from the PacBio HiFi platform provides advantages for identifying full-length transcripts and alternative splicing events, and have been widely used for gene annotation and investigation of transcriptome complexity in plants [19–22]. However, this technology has not yet been widely applied to *Citrus* genome annotation. These limitations impede in-depth investigations of gene structural variations, allelic diversity, and pangenomics in *Citrus* species.

To address these gaps, we generated a high-quality, haplotype-resolved telomere-to-telomere (T2T) genome assembly of pummelo (2*n* = 2*x* = 18) using both PacBio HiFi and Oxford Nanopore Technology (ONT) long-read sequencing data. We also developed a pipeline, HapGene, for accurate, unbiased gene annotation of haplotype-resolved genomes. By integrating hundreds of second-generation RNA-Seq and third-generation long-reads transcriptomic datasets, we used HapGene to conduct comprehensive annotations of protein-coding and non-coding genes for three key *Citrus* species: sweet orange and its two ancestral species, pummelo and mandarin. Further analyses included detailed characterization of resistance gene analogues (RGAs) and lincRNAs. Notably, the haplotype-resolved assembly and annotation allowed us to compressively characterize allelic gene divergence and allelic differentiation expression. Our study offers a valuable genomic resource and a novel genomic tool for the evolutionary and functional genomics, as well as genetic improvements of *Citrus* crops.

## Results

### Haplotype-resolved and telomere-to-telomere assembly of a pummelo genome

To obtain a haplotype-resolved assembly of pummelo, we sequenced the accession ‘GBY’ using PacBio HiFi and ONT ultra-long sequencing platforms, yielding 28.5 Gb of HiFi data and 50.7 Gb of ONT data. Based on the k-mer (21-mer) analysis of HiFi reads, the GBY genome is estimated to be ~321 Mb in size with a heterozygosity rate of 0.983% by GenomeScope [23] (Fig. S1). An initial assembly was constructed using hifiasm [23], which produced two haplotypes (GBY-hap1 and GBY-hap2) with total lengths of 348.96 and 347.20 Mb, contig N50 values of 38.58 and 32.57 Mb, and contig counts of 80 and 34, respectively. Subsequently, by aligning ONT ultra-long reads to the initial assembly, 13 contigs of GBY-hap1 and 12 contigs of GBY-hap2 were anchored to two sets of chromosome-scale scaffolds (9 each), each containing 4 and 3 gaps, respectively (Fig. S2). The final haplotype assemblies achieved total sizes of 342.54 and 344.04 Mb, with anchoring rates of 98.16% and 99.08%, respectively (Table S1).

**Figure 1:**
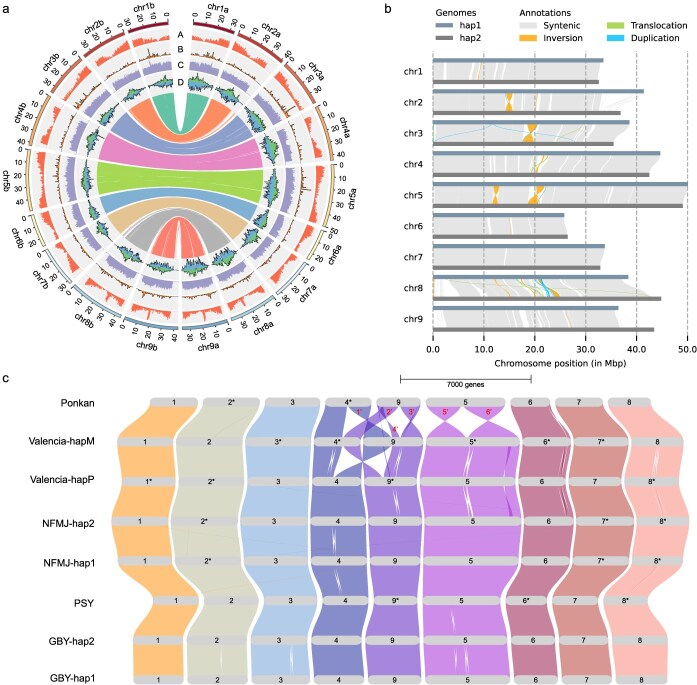
Haplotype-resolved and telomere-to-telomere assembly of a pummelo genome. (a) Genomic features of the two haplotypes GBY-hap1 (chr1a-chr9a) and GBY-hap2 (chr1b-chr9b) of the GBY (pummelo) genome. The outermost track indicates the nine chromosomes. Inner tracks (from outside to inside) represent gene density (A), lincRNA density (B), transposable element (TE) density (C), and Gypsy/Copia retrotransposon density (D). (b) Syntenic regions and structural variations between the two haplotypes of the GBY (pummelo) genome. (c) Gene macro-synteny across genomes of Ponkan [12] (mandarin), Valencia [12] (sweet orange), NanFengMiJu [28] (mandarin), PingShan [16] (pummelo) and GBY (pummelo). NFMJ: NanFengMiJu. PSY: PingShan pummelo. Scale bar, 7000 genes. Red numbers indicate the large structural variations (SVs) between Ponkan and Valencia-hapM, in which SVs 3, 5, and 6 are largely due to assembly artifacts, as revealed by Hi-C remapping analysis (Fig. S5). Chromosome numbers marked with an asterisk (*) indicate that the corresponding chromosome sequences are the reverse complements of the original sequences released in previous publications.

We employed multiple approaches to evaluate the quality of the genome assemblies. First, Benchmarking Universal Single-Copy Orthologs (BUSCO) [24] analysis showed high assembly completeness scores of 99.14% (GBY-hap1) and 99.63% (GBY-hap2) (Tables S1 and S2). The merged completeness score of two haplotype was 99.50%, exceeding that of most previously reported *Citrus* genomes and their wild relatives (Table S2). Second, base-level accuracy evaluation using Merqury [25] revealed quality values (QV) of 62 (GBY-hap1) and 64 (GBY-hap2) (Table S1), corresponding to nucleotide-level assembly accuracy of 99.99994% and 99.99997%. Third, the LTR Assembly Index (LAI) [26] values were 18.50 (GBY-hap1) and 18.14 (GBY-hap2), indicating that the assemblies of repetitive sequence regions meet reference-quality standards. Additionally, read remapping showed that 99.94% of HiFi reads aligned to GBY-hap1 and 99.98% to GBY-hap2; nearly 100% of ONT reads mapped to both assemblies, confirming full sequence representation (Table S1). We further used Inspector to evaluate the haplotype switch errors in the assembly based on alignments of both HiFi and ONT reads [27]. This analysis revealed 29 potential switch errors in GBY-hap1 and 26 in GBY-hap2, corresponding to regions of only 228 and 234 kb, respectively (Fig. S3), indicating a high level of phasing accuracy. Telomere characterization identified 15 complete telomeric regions in GBY-hap1 and 17 in GBY-hap2 (Table S3), verifying terminal coverage. Collectively, these results confirm the successful generation of a haplotype-resolved and T2T genome assembly (Fig. 1a).

Using GBY-hap1 as the reference, along with genome alignment tool minimap2 [29], and the sequence variation caller SyRI [30], we identified 292.06 Mb of syntenic regions in GBY-hap2, representing 84.9% of its total length. The analysis revealed extensive sequence variations between the two haplotypes, including 874 411 SNPs, 142 511 indels (1-50 bp), and structural variations (SVs; > 50 bp) comprising 3499 insertions, 3615 deletions, 35 inversions (10.56 Mb), 1212 duplications (3.56 Mb), and 997 translocations (3.21 Mb) (Fig. 1b and Table S4). The average variant density was 7.57 SNPs and 2.12 indels per kb, which was lower than that of the hybrid species sweet orange (Valencia genome: 15.25 SNPs and 2.85 indels per kb). The lower variant density in pummelo (a diploid progenitor species) compared to sweet orange (a hybrid) may reflect increased genomic heterozygosity associated with the hybrid origin of sweet orange. Among the SVs, four inversions exceeded 1 Mb in length, and their authenticity was further supported by HiFi read mapping patterns (Table S4 and Fig. S4). Notably, the terminal regions of chromosomes 2, 8, and 9 showed almost no collinearity between the two haplotypes due to the substantially high proportion of transposon elements (TEs) in these regions (Table S5), which was also supported by the collinearity analysis based on haplotype alignments generated by another repeat-tolerant tool (nucmer) (Table S6). These findings indicate substantial genomic divergence between the two haplotypes despite overall syntenic conservation.

In addition, synteny analysis of eight *Citrus* genomes, including two haplotypes each from GBY (pummelo), Valencia [12] (sweet orange), and NanFengMiJu [28] (mandarin), together with chromosome-level assemblies of Ponkan [12] (mandarin) and PingShan [16] (pummelo), confirmed widespread chromosomal collinearity (Fig. 1c). It is noteworthy that two pummelo genomes, one mandarin genome (NanFengMiJu), and Valencia-hapP exhibited collinearity at the two previously reported large inter-chromosome inverted translocation between chromosomes 4 and 9 of two haplotypes in Valencia [12]. However, another mandarin genome, Ponkan, presented additional large-scale rearrangements in chromosomes 5 and 9, most of which were assembly artifacts, as revealed by Hi-C remapping analysis (Fig. S5).

### The HapGene pipeline for haplotype-level gene annotation

**Figure 2:**
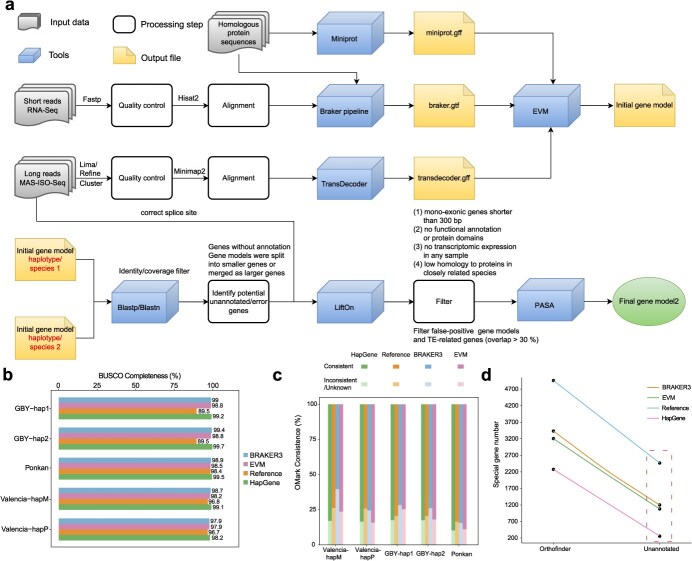
The HapGene pipeline for protein-coding gene annotation and its performance evaluation. (a) Overview of the HapGene pipeline. (b–c) Comparisons of annotation completeness and consistency between HapGene-derived annotations and annotations from other methods, using BUSCO (b) and OMark (c). (d) Comparisons of the number of haplotype-specific genes in the sweet orange (Valencia) haplotype Valencia-hapM (vs. Valencia-hapP) between HapGene-derived annotations and annotations from other methods. Orthofinder, indicates the number of initially identified haplotype-specific genes, based on Orthofinder-derived gene family clustering of protein sequences from Valencia-hapM and Valencia-hapP. Unannotated, indicates the potentially unannotated genes in the haplotype-specific gene set after excluding genes that only exist in one haplotype due to allele-specific sequence variations.

To fully leverage the haplotype assembly and improve the quality of gene annotation, we developed the HapGene pipeline specifically designed for haplotype-resolved assembly of heterozygous diploid genomes, to reduce false gene annotation differences between haplotypes, a limitation of traditional gene annotation methods. HapGene comprises two core steps: [1] initial annotation of each haplotype and [2] haplotype-aware annotation refinement (Fig. 2a; see the Materials and Methods for details). This approach implements a haplotype-aware annotation refinement strategy to address gene model discrepancies between haplotypes caused by incomplete or inconsistent preliminary annotations.

To evaluate the performance of HapGene, we assessed annotation completeness using two standard tools, BUSCO [24] and Compleasm [31] based on the two haplotype-resolved T2T assemblies of sweet orange (Valencia) and pummelo (GBY) genomes. For sweet orange, the BUSCO values of two haplotypes were 99.1% and 98.2% (Fig. 2b and Table S7), exceeding those from BRAKER3 (98.7%, 97.9%), EVM (98.2%, 97.9%), and the previously published annotation (96.8%, 96.7%) [12]. Similar improvements were observed for Compleasm scores. For pummelo, HapGene annotation also achieved high BUSCO (GBY-hap1: 99.2%, GBY-hap2: 99.7%) and Compleasm (GBY-hap1: 99.01%, GBY-hap2: 99.51%) scores (Fig. 2b and Table S7). Additionally, we used OMark [32], a specialized tool for assessing proteome quality, which not only evaluates gene completeness but also examines annotation consistency, detects potential contamination, and identifies gene redundancy. Compared to other annotations, HapGene annotations reduced the proportions of inconsistent annotation errors, or unknown genes were reduced compared to other annotations. For instance, in Valencia-hapM, HapGene annotations yielded 23 250 consistent genes (82.81%), 1173 inconsistent genes (4.18%), and 3653 unknown genes (13.01%), showing a marked improvement over annotations from BRAKER3 and EVM. (Fig. 2c and Table S8).

To test whether HapGene reduces false haplotype-specific genes (a common artifact of inaccurate annotation), we used OrthoFinder to cluster genes from each haplotype and identify haplotype-specific candidates. We detected 2275 and 2144 candidate haplotype-specific genes in sweet orange’s (Valencia) two haplotypes, and 1568 and 1485 in pummelo (GBY). After excluding potentially true haplotype-specific genes arising from heterozygous indels, partial/complete gene deletions or and heterozygous large-effect SNPs, the remaining genes were potential false ones. These included 257 and 146 genes for sweet orange, and 133 and 101 genes for pummelo, accounting for only ~1% of total genes. However, BRAKER3 and EVM based annotations yielded ~4% to 5% of false haplotype-specific genes (Fig. 2d and Fig. S6).

### Annotation of sweet orange, pummelo, and mandarin genomes

Next, we performed TE annotation for our GBY genome and 19 other previously published *Citrus* genomes (Tables S9 and S10). Specifically, GBY-hap1 and GBY-hap2 exhibited TE proportions of 50.75% and 52.01%, respectively (Table 1). Compared to sweet orange (e.g. Valencia-hapM: 44.96%, Valencia-hapP: 44.54%) and mandarin genomes (e.g. Ponkan: 49%), the pummelo GBY genome exhibited a slightly higher TE content, but lower than that of other pummelo genomes (e.g. Wanbai, 57.44%).

**Table 1:** Statistics of genome annotation

**Type**	**Valencia-hapM**	**Valencia- hapP**	**GBY- hap1**	**GBY- hap2**	**Ponkan**
**Percentage of TEs**	44.96%	44.54%	50.75%	52.01%	49.76%
**Protein-coding gene**					
Number of genes	28 076	27 665	27 433	27 485	25 162
Number of mRNAs	47 598	46 703	45 412	45 609	42 833
Number of genes with isoforms	7736	7577	7287	7345	7311
Mean isoform number per gene	1.7	1.7	1.7	1.7	1.7
**Non-protein-coding gene**					
Number of snRNA	68	83	73	78	76
Number of snoRNA	1465	1508	1614	1559	1637
Number of miRNA	154	152	146	151	159
Number of tRNA	422	504	913	482	569
Number of rRNA	762	874	1757	1670	264
Number of lincRNA	5363	5665	4100	4205	5892

**Figure 3:**
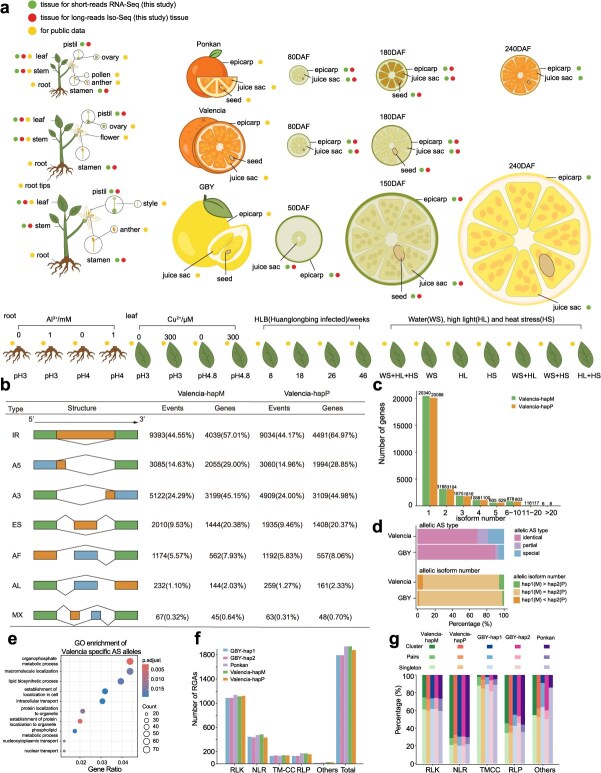
Gene annotation of sweet orange (Valencia), pummelo (GBY) and mandarin (Ponkan). (a) Schematic diagram of sampled tissues for RNA-Seq and Iso-Seq from this study and previous studies. Colored dots indicate the source of transcriptome data across diverse tissues, developmental stages, and sampling conditions. DAF: Days After Flowering. (b) Summary of alternative splicing (AS) differences between the two haplotypes of the sweet orange (Valencia) genome. IR: intron retention, A5: alternative 5′ splice-site, A3: alternative 3′ splice-site, ES: exon skipping, AF: alternative first exon, AL: alternative last exon and MX: mutually exclusive exons. (c) The distribution of isoform numbers per gene in the two haplotypes of the sweet orange (Valencia) genome. (d) The proportions of different allelic AS types and allelic isoform number in the sweet orange (Valencia) and pummelo (GBY) genomes. Allelic AS types were classified as: identical (completely shared AS types between haplotypes), partial (partially shared AS types), and special (completely distinct AS types between haplotypes). (e) GO enrichment analysis of genes (special) with differential AS. (f) Summary of resistance genes analogues (RGAs) classified into subclasses (RLK, RLP, NLR, TM-CC, and Others). (g) Percentages of different RGA types classified into Singleton, Pairs, and Cluster groups.

Apart from T2T assemblies, we aimed to improve the gene annotation of *Citrus* genomes using the HapGene pipeline. We selected three representative *Citrus* species, sweet orange (*C. sinensis*) and its two hybrid ancestors, pummelo (*C. maxima*) and mandarin (*C. reticulata*). Three corresponding accessions, Valencia, GBY, Ponkan were selected for Iso-Seq (PacBio HiFi platform), and short-read RNA-Seq (Illumina platform). For Iso-Seq, we pooled 27 RNA samples from seven different tissues per accession (Fig. 3a), generating 9.46 to 12.54 Gb of long-read transcriptome sequencing data per accession (Table S11). For RNA-Seq, we generated 22 new datasets covering seven tissues (including epicarp and juice sac at multiple developmental stages) (Fig. 3a), yielding a total of 347.32 Gb (Valencia, 59.44 Gb; GBY, 144.14 Gb; Ponkan, 143.74 Gb) reads (Table S11). Additionally, we integrated 364 publicly available RNA-Seq datasets representing 17 tissues and 19 conditions (Fig. 3a), totaling 1984.71 Gb for sweet orange, 323.50 Gb for pummelo, and 484.02 Gb for mandarin (Table S12). Iso-Seq generated 7.67, 5.24, and 5.45 million reads for Valencia, GBY, and Ponkan, respectively. The average read length, N50, and proportion of full-length non-chimeric (FLNC) reads were comparable across the three accessions, with >99% of transcripts containing both primers and poly(A) tails (Table S13).

For the two haplotypes of sweet orange, we annotated 28 076 and 27 665 protein-coding genes, respectively, with 86.89% and 85.88% supported by long or short RNA-Seq reads (Table 1) and the average gene length of 3.05 and 3.16 kb (Fig. S7). Similar counts of genes were annotated for both haplotypes (27 433 and 27 485) of pummelo, while relatively fewer genes for the mandarin genome (25 162 genes). We identified a total of 21 083 and 20 452 alternative splicing (AS) events in sweet orange’s haplotypes, encompassing intron retention (IR), alternative 5′ splice-site (A5), alternative 3′ splice-site (A3), exon skipping (ES), alternative first exon (AF), alternative last exon (AL) and mutually exclusive exons (MX) (Fig. 3b). IR was the most frequent type (44.55% and 44.17% of total events), present in 8620 and 8265 transcripts (47.21% and 46.62%). Pummelo's two haplotypes and mandarin had 19 228, 19 392, and 18 751 AS events, respectively, with similar IR-dominant patterns (48.36%, 48.57%, and 43.47% IR) (Fig. S8).

AS events produced 47 598 (hapM, mandarin-origin) and 46 703 (hapP, pummelo-origin) isoforms in sweet orange's haplotypes, with similar isoform distributions per gene (Fig. 3c). On average, each gene has 1.7 isoforms for the two haplotypes. Most (72.45% for hapM, 72.61% for hapP) genes only contained one isoform, while 0.43% and 0.45% had more than ten. For example, *CsiH1chr6_g178400* (Valencia-hapM, chr6: 3204878–3 211 247) encoded 14 isoforms (Fig. S9). Pummelo and mandarin showed analogous isoform patterns (Fig. S10a and b).

We further compared allelic alternative splicing (AS) event types and allelic divergence in isoform number between haplotypes. In sweet orange, among 6477 allelic gene pairs (defined as pairs with AS events in at least one of the two haplotypes), 4500 (69.48%) showed identical AS event types (Fig. 3d). Gene ontology (GO) enrichment analysis of genes with haplotype-specific AS event types revealed significant enrichment in biological processes like organophosphate metabolic process and lipid biosynthetic process, suggesting potential roles in metabolic regulation, cellular organization, and transport activities (Fig. 3e). In terms of isoform number, 1253 hapP genes (6.69%) and 1346 hapM genes (7.18%) contained more isoforms than their allelic counterparts (Fig. 3d). By contrast, the pummelo showed lower allelic divergence, which was consistent with the closer phylogenetic relationship between its two haplotypes (Fig. 3d and Fig. S11). Specifically, among 6754 allelic gene pairs (defined as in sweet orange), 6081 (90.04%) shared the identical AS event types, and 21 943 (95.88%) allelic genes had the same number of isoforms between haplotypes (Fig. 3d).

Moreover, we developed a workflow to annotate resistance gene analogues (RGAs), including receptor-like protein kinases (RLKs), nucleotide-binding leucine-rich repeat receptors (NLRs), transmembrane coiled-coil (TM-CC) proteins, receptor-like proteins (RLPs), and others with only TIR or RPW8 domains (see Materials and Methods). These five haplotypes (two each for sweet orange/pummelo, one mosaic for mandarin) contained 1725–1853 RGAs (8946 in total), in which RLKs occupied the majority (57.00%–60.11%) (Fig. 3f and Table S14), followed by NLR and RLP. Furthermore, homologous gene clustering of RGAs across these five haplotypes identified 972 core gene families (present in all five haplotypes) and 554 variable gene families (absent in at least one haplotype). These core families encompassed 6573 RGAs (73.47%), implying that the majority of RGA genes were conserved among *Citrus* species (Fig. S12). We also identified 96 potentially species-specific RGAs in sweet orange, 96 in pummelo, and 32 in mandarin—genes that may lay the foundation for species-specific defense against biotic or abiotic stresses.

These RGAs were classified into singletons, pairs, and clusters based on chromosome distribution and intergenic distance, showing distinct distribution patterns (Fig. 3g and Table S14). Notably, NLRs and RLPs had higher proportions (67.55%–71.34% and 45.00%–56.76%, respectively) of clustered genes across all haplotypes compared to other gene families. This clustering pattern of RGA genes is consistent with their roles in plant defense, where tandem duplication and local expansion drive rapid gene family diversification [33].

### Characterization and expression analyses of lincRNAs

In addition to protein-coding genes, we annotated various classes of non-coding RNAs, including transfer RNAs (tRNAs), ribosomal RNAs (rRNAs), small nuclear RNAs (snRNAs), small nucleolar RNAs (snoRNAs), microRNAs (miRNAs) and long non-coding RNAs (lncRNAs). As summarized in Table 1, the number of annotated non-coding RNA genes varied across genomes of sweet orange, pummelo and mandarin, with tRNAs ranging from 422 to 913, rRNAs from 264 to 1757, and lncRNAs from 4100 to 5892. The annotation also identified 68 to 83 snRNAs, 1465 to 1637 snoRNAs, and 146 to 159 miRNAs.

**Figure 4:**
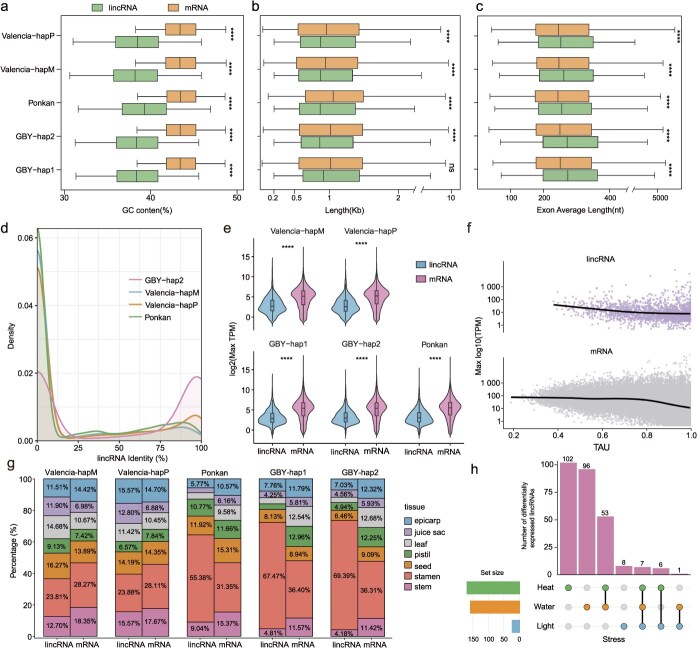
Characterization and expression analysis of lincRNAs across the genomes of sweet orange (Valencia), pummelo (GBY), and mandarin (Ponkan). (a-c) Comparisons of GC content (a), transcript length (b), and average exon length (c) between lincRNAs and mRNAs. (d) Density distribution of homologous lincRNA sequence identities between the reference genome (GBY-hap1) and other genomes. (e) Comparisons of transcript expression levels (TPM) between lincRNAs and mRNAs. Statistical significance was determined by the Wilcoxon rank-sum test (^****^P < 0.0001). (f) Relationship between tissue specificity (TAU) and gene expression levels for lincRNAs (top) and mRNAs (bottom) in pummelo (GBY-hap1). Each point represents a gene. Solid lines indicate locally fitted regression curves. (g) The percentage of tissue-specific lincRNAs with dominant expression in each tissue. A lincRNA was defined as tissue-specific if the ratio of its highest to second-highest expression levels (TPM) exceeded 3. (h) Upset plot showing the number and intersections of differentially expressed lincRNAs (either upregulated or downregulated) in sweet orange (Valencia-hapM) under heat, light, and water stress conditions. Differential expression was determined using |log₂(fold change)| > 1 and false discovery rate (FDR) < 0.05.

To gain a deeper understanding of the roles and genomic characteristics of long intergenic non-coding RNAs (lincRNAs), we used Plant-LncPipe [34] to integrate full-length third-generation transcriptomic data and RNA-Seq data for systematic lncRNA annotation. LncRNAs were then classified based on genomic locations, with those mapping to intergenic regions (and non-overlapping with annotated protein-coding genes) designated as lincRNAs. In total, we annotated 15 061 lincRNAs across the three genomes: 3052 (Valencia-hapM), 3392 (Valencia-hapP), 2392 (GBY-hap1), 2468 (GBY-hap2), and 3757 (Ponkan) (Table 1). Compared to a previous *Citrus* study [35], our lincRNA set showed a 12.25% to 24.75% increase in sweet orange, 10.38% to 13.89% increase in pummelo, and 174.84% increase in Ponkan. Among the annotated lincRNA, 34.91%–46.58% were supported by full-length long-read transcripts. These lincRNA presented similar patterns of GC content, transcript length, and average exon length between haplotypes and species. Relative to mRNA, they exhibited shorter transcript lengths and lower GC content, but longer average exon lengths (Fig. 4a–c). These structural differences likely reflect distinct functional constraints and evolutionary pressures, consistent with observations in Brassicaceae and suggesting conserved lincRNA features across plant lineages [36].

On average, 34.5% of lincRNAs overlapped with transposon elements (TEs) more than 50% of their regions, suggesting that TEs may play a key role in lincRNA origination (Fig. S13). Among TE-related lincRNAs, long terminal repeat (LTR) retrotransposons and DNA transposons were the dominant TE types (Fig. S14). Such overlapping with TEs indicated they might show relatively higher sequence diversity. This could be inferred from the inter-species sequence comparisons, where lincRNAs showed lower sequence similarity compared to mRNA (Fig. 4d and Fig. S15). This was also for the lincRNA between haplotypes in the same genomes, such as pummelo, where a majority (53.30%) of lincRNA presented sequence identity lower than 80%.

Subsequently, we conducted a comparative analysis of the overall expression levels of mRNAs and lincRNAs, as well as their tissue-specific expression patterns. Overall, mRNAs exhibited substantially higher expression levels than lincRNAs, with the maximum expression of mRNAs in each tissue exceeding threefold that of lincRNAs (Fig. 4e). In GBY-hap1 (pummelo), we systematically analyzed the relationship between tissue specificity and gene expression levels by plotting tissue specificity (TAU) values [37] against expression. The results showed that lincRNAs generally exhibited higher tissue specificity, whereas mRNAs displayed broader expression patterns across tissues (Fig. 4f). Interestingly, both GBY and Ponkan displayed a pattern similar to that observed in mammals, with lincRNAs preferentially expressed in male reproductive tissues (Fig. 4g). Our results indicate that lincRNA expression varies markedly across tissues, with this specificity providing important clues about their functional roles in diverse biological scenarios. Beyond tissue-dependent regulation, their stress-responsive patterns also point to potential involvement in environmental adaptation. We therefore further analyzed lincRNA differential expression under heat, light, and water stress based on public RNA-Seq datasets from sweet orange (Fig. 4h). Across these stress treatments, we identified 273 significantly differentially expressed lincRNAs. Among of them, 53 were shared between heat and water stresses, whereas far fewer were shared between light stress and the other two. This suggests that many lincRNAs may play divergent roles in responding to different types of stress (Fig. 4h).

### Allelic gene differentiation in sweet orange and pummelo

**Figure 5:**
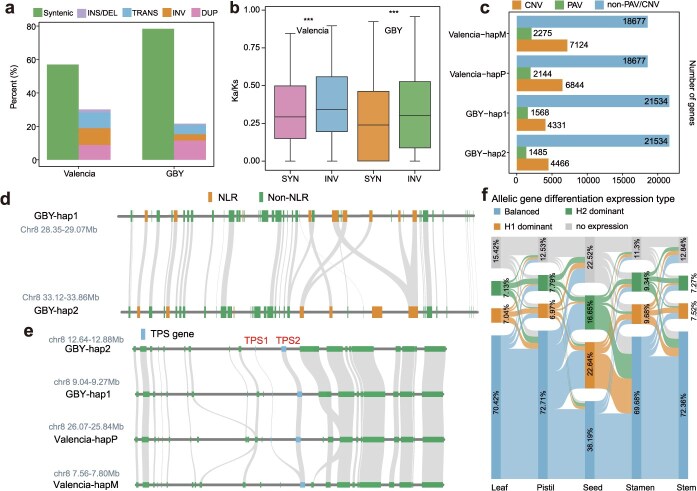
Allelic gene differentiation in sweet orange and pummelo. (a) The average proportion of syntenic and rearranged genes across pairwise haplotype sequence alignments in pummelo (GBY) and sweet orange (Valencia) genomes. Rearranged regions include insertions (INS), deletions (DEL), duplications (DUP), inversions (INV), and translocations (TRANS), identified via whole-genome alignments. (b) Comparison of Ka/Ks ratios for allelic gene pairs between syntenic (SYN) and inverted (INV) regions Valencia and GBY. Statistical significance was determined using the Wilcoxon rank-sum test (^***^P < 0.001). (c) Classification of genes into PAV, CNV, and non-PAV/CNV categories in Valencia and GBY. (d, e) Representative examples of allelic gene differentiation driven by structural variations. (d) A region on chromosome 8 of GBY (pummelo) exhibiting both PAV and CNV of NLR genes between haplotypes. (e) A region on chromosome 8 showing TPS gene CNV across pummelo (GBY) and sweet orange (Valencia) haplotypes. (f) Sankey plot showing the proportion of allelic expression patterns including Balanced, H1_dominant, H2_dominant, and no expression across different tissues in GBY (pummelo). Balanced: equal expression of both alleles, H1_dominant: significantly higher expression of haplotype 1, H2_dominant: significantly higher expression of haplotype 2, and NE: no expression.

The haplotype-resolved T2T assemblies of sweet orange and pummelo allowed us to characterize the gene-level haplotype divergence including presence/absence variation (PAV) and copy number variation (CNV). Compared to sweet orange, pummelo harbored far more genes within syntenic regions between its two haplotypes (Fig. 5a). Genes located in INV regions exhibited significantly higher Ka/Ks ratios compared to those in syntenic regions (Fig. 5b). The higher Ka/Ks ratios in inverted regions may reflect reduced recombination rates (due to structural rearrangement), weakening purifying selection and facilitating accumulation of adaptive mutations [33]. SVs and small variants together resulted in PAVs and CNVs of genes between haplotypes. In total, 4419 and 3053 PAV genes, as well as 13 968 and 8797 CNV genes were identified between haplotypes in sweet orange (Valencia) and pummelo (GBY) genomes, respectively (Fig. 5c). This higher abundance of PAV and CNV genes in Valencia is consistent with its hybrid origin (derived from pummelo and mandarin).

CNVs genes were significantly enriched in GO terms related to ADP binding and defense response (Fig. S16). For example, in an allelic region (28.35–29.07 and 33.12–33.86 Mb) on chromosome 8 of GBY, multiple NLR genes exhibited both PAV and CNV between haplotypes, indicating substantial structural divergence that may underpin haplotype-specific immune responses (Fig. 5d). Additionally, CNVs were also observed in genes associated with secondary metabolism like terpenoid biosynthesis. For instance, two tandemly duplicated TPS copies located on two chromosome 8 (Chr8:9.04–9.27 and 12.64–12.88 Mb) of GBY and Valencia-hapP (Chr8:25.84–9.27 Mb). In contrast, Valencia-hapM harbored only one TPS copy (Chr8:7.56–7.80 Mb), indicating haplotype-specific contraction that may affect terpenoid metabolism (Fig. 5e).

Based on homology and collinearity analyses, we identified 18 737 and 22 887 bi-allelic loci in the Valencia and GBY genomes, respectively. To investigate how allelic variation impacts expression, we performed differential expression analysis between bi-allelic genes using RNA-Seq data from multiple tissues of both species. After removing bi-alleles that are not expressed in any tissue and those with completely identical sequences, 17 081 (Valencia) and 16 280 (GBY) bi-alleles were categorized into four expression categories: Balanced (equal expression of both alleles), H1_dominant (significantly higher expression of haplotype 1), H2_dominant (significantly higher expression of haplotype 2), and NE (no expression). A Sankey diagram visualized dynamic transitions in allelic expression states across tissues and fruit developmental stages (Fig. 5f and Fig. S17). This classification revealed that 31.47% of Valencia and 35.33% of GBY bi-alleles demonstrated divergent allelic expression patterns across different tissues, implying that allelic specific sequences or regulatory elements might control the allele-specific expression under different tissues. Notably, balanced-expression alleles accounted for less than 40% in GBY seeds, with haplotype-specific expression significantly enriched to other tissues, implying a distinct regulatory mechanism governing allele-specific expression in seeds. Genes with seed-specific allelic expression patterns divergent from other tissues were significantly enriched in GO terms, such as transferase complexes, mRNA binding, and intracellular protein-containing complexes, suggesting their involvement in seed-specific regulatory processes (Fig. S18).

## Discussion

Haplotype-resolved T2T assemblies facilitate comprehensive exploration of allelic sequence and expression divergence in both canonical genomic regions and hard-to-access regions, which further enables the pinpointing of trait-associated allele-specific variants and improve gene editing efficiency in heterozygous regions [5–7]. Here, we report the first haplotype-resolved T2T genome for pummelo, representing substantial improvements in continuity, completeness, and haplotype resolution. This high-quality resource allowed us to identify extensive allelic variants and differential expression, laying a foundation for haplotype-aware genomic analyses of speciation and crop domestication. Notably, extensive interspecific hybridization and genetic introgression are prevalent among *Citrus* and *Citrus*-related species [9, 10], resulting in complex genomic architectures that have obscured the origin and evolutionary history of *Citrus*. Previous studies have remained inconclusive on these key questions, largely due to the lack of high-quality haplotype-resolved assemblies [9, 10]. For example, accurate genomic analyses to unravel pummelo's origin and domestication history are challenging due to pervasive genetic introgression [38]. Our pummelo assembly, together with future expanded haplotype-resolved T2T resources, will facilitate the disentanglement of the complex evolutionary processes (e.g. origin, admixture, and phylogeny) of pummelo and other *Citrus* crops [28, 39], thereby advancing genomics-assisted breeding for trait improvement.

Apart from the lack of high-quality assemblies, the quality of gene annotation in *Citrus* remains limited, which hinders functional genomic studies. BUSCO-based completeness evaluation revealed there were substantial unannotated genes (4.98% on average) in 45 published *Citrus* and their relatives' genomes (Table S2). This limitation stems primarily from short-read RNA-Seq data constraints and inherent flaws in conventional annotation pipelines. The long read based transcriptome sequencing technologies, including HiFi-based Iso-Seq and ONT based Direct RNA Sequencing (DRS) [40], can capture full-length transcripts, potentially providing accurate splicing junction information [19, 22, 41]. They have been widely applied for detangling transcriptome complexity and improving gene annotation. However, their application in *Citrus* has been limited [42–44]. Here we generated Iso-Seq data for three *Citrus* species, which significantly improve gene annotation quality and enabled comprehensive characterization of alternative splicing events. Additionally, integrating this data with a large corpus of published multi-tissue RNA-Seq datasets provided valuable resources for updating gene models and profiling expression [45]. This integrative approach yielded highly complete and accurate annotations of both protein-coding and non-coding genes across three *Citrus* species.

Notably, we identified 4.48–9.52% novel protein-coding genes, and additional 10.38–174.84% lincRNAs compared to previous annotations. Moreover, the large number of lincRNAs associated with transposable elements provides novel insights into mechanisms of stress response and epigenetic regulation in *Citrus*. Beyond protein-coding genes and lincRNAs, our high-quality assembly also enables refined characterization of resistance-related genes. Similar to observations in other plant species [33, 46], resistance genes, especially NLRs, in *Citrus* genomes tend to form clusters, a pattern attributed to co-evolution with pathogens and functional optimization [47]. This clustering is typically driven by tandem duplication and transposon-mediated replication, which thereby expand NLR gene families and provides genetic material for diversification [46, 47]. Functionally, genes within NLR clusters can cooperate to mediate efficient defense responses [48]. More haplotype-resolved T2T assemblies will further facilitate the precise structural and evolutionary characterization of RGA clusters across distinct haplotypes, eliminating ambiguity associated with collapsed genome assemblies.

Remarkably, we developed HapGene, a new annotation pipeline tailored for haplotype-resolved diploid genome assemblies. Most previous diploid genome projects annotate each haplotype separately. However, even with the identical workflow, annotation between haplotypes still contain spurious differences such as false haplotype-specific genes due to the incomplete annotation or erroneous gene structures in one haplotype. By implementing haplotype-aware refinement of primary gene models, HapGene substantially reduces these artifacts, enabling precise distinction between hemizygous and bi-allelic genes, compared to other state-of-the-art annotation approaches such as BRAKER3 and EVM. Further iteration of HapGene could be adapted for polyploid genomes, and pangenomes where independent per-genome annotation often generates false lineage-specific genes and distorts pangenome gene repertoires.

Overall, this study delivers high-quality genomic resources and a scalable annotation framework for heterozygous species, laying a foundation for targeted gene editing and the development of high-resolution molecular markers for genomics-assisted breeding [4, 7]. Future research should integrate multi-omic data (transcriptomic, proteomic, epigenomic, and regulomic) to fully dissect the structure and function of coding and non-coding genes. Expanding haplotype-resolved T2T assemblies and annotations to diverse wild *Citrus* germplasms will further advance our understanding of *Citrus* genome evolution and crop domestication, while furnishing critical resources to support *de novo* domestication and rational haplotype design [12, 49].

## Materials and methods

### Plant material and sample preparation

In this study, three *Citrus* species were selected: sweet orange (*Citrus sinensis* cv ‘Valencia’), pummelo (*Citrus maxima* cv ‘GBY’), and mandarin (*Citrus reticulata* cv ‘Ponkan’). All plant materials were obtained from the National Citrus Breeding Center at Huazhong Agricultural University in Wuhan, China.

Genomic DNA of pummelo was extracted from young leaves for genome sequencing. Total RNA of three *Citrus* species was extracted from seven tissue types including pistil, stamen, stem, leaf, epicarp, juice sac, and seed. Among them, epicarp and juice sac were sampled at two fruit developmental stages. All samples were subjected to RNA-Seq using the Illumina platform. Specifically, for Valencia and Ponkan, epicarp and juice sac samples were collected at 80 and 180 days after flowering (DAF 80 and DAF 180), while for GBY, the corresponding samples were collected at DAF 50 and DAF 150.

### Genome and transcriptome sequencing

For PacBio HiFi genome sequencing, genomic DNA was extracted using the CTAB method, purified with the Grandomics Genomic kit, and used to construct SMRTbell libraries with the SMRTbell prep kit 3.0 (PacBio, USA). Libraries were sequenced on the PacBio Revio platform at Grandomics Biosciences Co., Ltd (Wuhan, China). For Oxford Nanopore Technologies (ONT) genome sequencing, genomic DNA was extracted using the Grandomics DNA kit for regular libraries and the BAC-long DNA kit for ultra-long libraries. DNA quality and quantity were evaluated with NanoDrop™ One (Thermo Fisher Scientific, USA) and Qubit® 3.0 Fluorometer (Invitrogen, USA). High-molecular-weight fragments were size-selected using the PippinHT/BluePippin system (Sage Science, USA). DNA libraries were prepared with the SQK-LSK114 kit (ONT, UK) and sequenced on the PromethION platform using R10.4.1 flow cells under the SUP model (25 kHz, 400 bps, sup@v4.2.0) at Grandomics Biosciences (Wuhan, China).

For Illumina short-read transcriptome sequencing, mRNA libraries were constructed by enriching poly(A) + RNA using oligo(dT) magnetic beads, followed by controlled fragmentation and double-stranded cDNA synthesis. After end repair, A-tailing, and ligation of indexed sequencing adapters, adapter-ligated fragments were size-selected and purified with paramagnetic beads. The libraries were then PCR-amplified, purified, and assessed for quality using a Qsep-400 fragment analyzer before sequencing. For full-length transcriptome sequencing, libraries were constructed using the PacBio Kinnex Iso-Seq protocol. Tissue-specific RNA was extracted, pooled, and used for library preparation with the Iso-Seq Express 2.0 kit (PacBio, USA). Sequencing was carried out on the PacBio Revio platform (Grandomics Biosciences, Wuhan, China).

### The T2T genome assembly of the pummelo (GBY) genome

A total of 28.5 Gb HiFi and 50.7 Gb ONT long reads were used to generate the initial haplotype-resolved assembly using hifiasm v0.19.8-r603 [50]. Based on alignment information between ONT reads and the initial assembly, a chromosome-level genome was constructed by iteratively filling gaps with ONT reads. To improve base-level accuracy, the resulting assembly was further polished with NextPolish2 [51] by integrating PacBio HiFi and Illumina reads. The LTR Assembly Index (LAI) was calculated using LTR_retriever v3.0.1 [52]. Circos v0.69–8 [53] was used to visualize genome features. Sequence variations between haplotypes were identified using SyRI v1.6.5 [30]. GENESPACE v1.3.1 [54] was employed to detect synteny among the GBY, Ponkan (mandarin), and Valencia (sweet orange) genomes and to generate the riparian plot for displaying gene-level synteny.

### The HapGene pipeline

To enable accurate and consistent gene annotation in heterozygous diploid genomes, we developed an annotation pipeline, HapGene, which integrates multi-source evidence and implements haplotype-aware refinement strategies. The first step involves initial annotation of each haplotype using ab initio gene prediction, homology-based inference, and transcriptomic evidence.

#### Initial annotation of each haplotype

Specifically, short-read RNA-Seq data are preprocessed using fastp v0.23.4 [55] for adapter removal and quality trimming, aligned to each haplotype assembly using Hisat2 v2.2.1 [56], and alignments with mapping quality scores below 30 are removed using samtools v1.12 [57]. The remaining reads are sorted and subsequently used as input for the BRAKER3 [58] annotation pipeline including Augustus [59] and GeneMark-ETP [60].

For the long-read Iso-Seq data generated by the PacBio HiFi sequencing platform, the Iso-Seq pipeline (https://www.pacb.com/products-and-services/analytical-software/isoseq/) is employed for data preprocessing. In detail, lima is used to remove 5′ and 3′ primer sequences, followed by refine to eliminate chimeric reads and trim polyA tails. The resulting reads are then clustered using cluster tools to obtain FLNC transcripts. The FLNC reads are aligned to the reference genome using Minimap2 v2.28-r1209 [29], and the alignment results are filtered and sorted using samtools. Transcript assembly is performed using StringTie v2.2.1 [61, 62] with the -L parameter to leverage the long-read alignment data. TransDecoder v5.5.0 [63] is then used to identify candidate coding regions by extracting long open reading frames (ORFs) and predicting potential coding sequences. The cdna_alignment_orf_to_genome_orf.pl script from TransDecoder is employed to generate genome-based gene models from the full-length transcript data.

To enhance the accuracy of gene annotations, homologous protein sequences from model species and closely related species are used for homology-based prediction. Gene models predicted from the three strategies including RNA-Seq data, long read transcriptome data, and homology evidence are integrated using EvidenceModeler (EVM) v2.1.0 [64] to produce the preliminary annotation. During evidence integration, predictions derived from long-read transcriptome data are assigned the highest weight, as third-generation sequencing typically provides superior structural accuracy.

#### Haplotype-aware annotation refinement

After obtaining the preliminary annotation, correction and refinement are performed. First, splice site information is extracted from the third-generation full-length transcriptome data using the bam2hints.pl script to identify accurate exon-intron junctions. For each species with two haplotypes, gene sequences from one haplotype are aligned to the other haplotype’s genome using BLAST v2.14.1 + [65] and alignments with low identity (<80%) or coverage (<80%) are filtered out. In regions where no gene models are present, homologous proteins aligned to these regions are used for further annotation, with corrections informed by splice site evidence to resolve missed genes. Similarly, protein sequences from one haplotype are aligned to those of the other haplotype using BLAST v2.14.1 + [65] or diamond v2.1.9 [66], and only alignments with identity and coverage ≥80% are retained. If a single protein sequence aligned to two adjacent predicted proteins, or if two adjacent protein sequences aligned to a single protein, the region is further examined using splice junction information and read coverage data to correct potential split or merged gene structure errors. In addition, the LiftOn v1.0.5 [67] tool is used to transfer annotations, ensuring consistency and accuracy in genome annotation (Fig. 2a). Subsequently, transposable element (TE)-related genes, defined as those overlapping annotated TEs by more than 30%, were removed. Genes that simultaneously met all four of the following criteria were then considered putative false positives and excluded: [1] mono-exonic genes shorter than 300 bp; [2] lacking functional annotation or identifiable protein domains; [3] not expressed in any samples; and [4] exhibiting low homology to proteins in closely related species. Finally, untranslated regions (UTRs) were incorporated using PASA to generate the final gene models.

### Protein-coding gene annotation of sweet orange, pummelo, and mandarin

Apart from RNA-Seq and Iso-Seq data sequenced in this study, we additionally collected 1984.71 Gb, 323.50 Gb, 484.02 Gb RNA-Seq from different tissues for sweet orange, pummelo, and mandarin. Details of these datasets were described in the Table S12. Homologous protein sequences from *Arabidopsis thaliana*, *Oryza sativa*, and closely related *Citrus* species such as *Citrus ichangensis* (Table S15) were also collected. Protein-coding genes were annotated using the HapGene pipeline by integrating these transcriptomic and protein sequence data as described above.

### Evaluation of gene annotation

We evaluated the completeness of genome annotation using BUSCO/Compleasm with the embryophyta_odb10 dataset, including 1614 single-copy orthologs conserved across land plants. Additionally, OMark v0.3.1 [32] was employed to assess the consistency and accuracy of annotation results.

To systematically evaluate the concordance and reliability of gene annotations between the two haplotypes, we developed an integrated framework that combines ortholog clustering and structural variation analysis for the identification and validation of haplotype-specific genes. We first applied OrthoFinder to perform whole proteome ortholog clustering for both haplotypes. Cluster with genes from only one haplotype, are initially considered as candidate haplotype-specific genes. However, such candidates may arise from annotation biases, assembly or clustering artifacts, or haplotype sequence differences. To this end, we used SyRI v1.7.0 [30] to detect variations between the two haplotypes.

Subsequently, we used SnpEff v5.2a [68] to annotate the functional effects of sequence variants. High effect variants typically represent disruptive mutations including loss of start codons or stop codons, premature stop codons, frameshifts, loss of splicing sites. These variants are likely to result in loss of gene function. Based on this annotation, we considered candidate genes to be high-confidence haplotype-specific if they met any of the following criteria: (i) they were annotated as HIGH effect by SnpEff; (ii) they were located within regions affected by SVs (≥50 bp) which directly delete the whole gene or partial coding regions (require at least 50%), insert a long sequence in coding region, or invert the coding region; (iii) they resided in unaligned genomic regions (defined by SyRI). Finally, the difference between these high-confidence haplotype-specific genes and OrthoFinder based haplotype-specific genes were defined as candidate unannotated genes due to annotation errors. Notably, residual errors from assembly or OrthoFinder clustering may also introduce a few unannotated genes.

### Annotation of non-coding RNA

To achieve a thorough and precise annotation of non-coding RNAs (ncRNAs), we integrated multiple prediction approaches, each optimized for specific ncRNA categories. These include transfer RNAs (tRNAs), ribosomal RNAs (rRNAs), small nuclear RNAs (snRNAs), small nucleolar RNAs (snoRNAs), and microRNAs (miRNAs). The tRNA identified by tRNAscan-SE v2.0.12 [69]. The rRNA were annotated with RNAmmer v1.2 [70]. Other classes of small non-coding RNAs, such as snRNAs, snoRNAs, and miRNAs, were identified using Infernal v1.1.5 [71] in combination with the Rfam covariance model database.

### Repeat annotation

For repetitive element annotation, we used EDTA v2.2.2 [72] to construct a de-novo transposable element (TE) library. EDTA integrates tools such as RepeatModeler [73], LTR_retriever [52] and RepeatMasker [74] to comprehensively identify major TE types including LTRs, TIRs, and Helitrons. The pipeline automatically removes redundancy and generates a high-quality TE library. This library was then used as a reference by RepeatMasker v4.1.2-p1 (https://www.repeatmasker.org/) to annotate repetitive sequences across the whole genome.

### Annotation of resistance gene analogues

Resistance gene analogues (RGAs) include receptor-like protein kinases (RLKs), nucleotide-binding leucine-rich repeat receptors (NLRs), transmembrane coiled-coil (TM-CC) proteins, receptor-like proteins (RLPs), and other potential RGAs with only TIR or RPW8 domains. For RLKs, we downloaded two canonical kinase domains HMM profiles from the Pfam database (PF07714 and PF00069), along with their corresponding seed sequences. Candidate genes were first identified by combining results from HMMER (hmmsearch) [75] and BLASTP, followed by redundancy removal. Based on the method described by Lehti-Shiu and Shiu [76], we further constructed custom HMMs using the kinase sequences provided in the study to refine RLK family classification. To ensure structural integrity, we applied deepTMHMM v1.0 [77] to predict transmembrane (TM) domains and excluded transcripts containing more than two TM domains, resulting in a high confidence set of RLK genes.

For the NLR (nucleotide-binding leucine-rich repeat) resistance gene family, we first performed domain annotation on all predicted proteins using InterProScan v5.74 [78]. This allowed the detection of key NLR-associated domains such as TIR, CC, and RPW8. Next, we applied NLR-Annotator v2.0 [79] to annotate and classify candidate NLR genes. As NLR-Annotator does not detect RPW8 domains, we supplemented its results by manually including genes containing both RPW8 and NB-ARC domains, as identified by InterProScan, and categorized them as RN-type NLRs. All final NLR genes were categorized based on their domain compositions. Additionally, we employed the RGAugury pipeline [80] to identify other resistance gene analogs (RGAs), including RLPs and TIR-only genes lacking NBS domains.

### Allele identification and expression analysis

To identify allelic gene pairs between different haplotypes, we adopted a strategy integrating both collinearity and homology information. First, we performed genome-wide collinearity analysis using MCScan (JCVI Python version) [81], retaining high-confidence collinear gene pairs located in ‘lifted anchor’ regions with the parameter jcvi.compara.catalog ortholog —cscore = 0.99. Next, homologous gene relationships were inferred using OrthoFinder v2.5.4, and collinear gene pairs were filtered based on their membership in the same homologous group. For genes with multiple potential matches, all candidate pairs were retained, and the one with the highest collinearity score was designated as the effective allele. Gene pairs present in both haplotypes were classified as bi-allelic, while genes uniquely present in a single haplotype were considered mono-allelic. Finally, gene pairs with no allelic differences were removed to retain only divergent allele pairs for downstream analyses.

Allelic TPM values were quantified using kallisto v0.51.1 [82] and normalized to the total gene-level expression. Each expression pattern was assigned to one of four categories: H1_dominant, H2_dominant, Balanced, or NE (Not Expressed), based on the principle of minimizing the Euclidean distance between the observed expression profile and predefined ideal vectors.

### Ka/Ks analysis

To investigate evolutionary dynamics across different gene categories, including PAV, CNV, core, and dispensable genes, we calculated nonsynonymous (Ka) and synonymous (Ks) substitution rates using a two-step strategy. First, we employed ParaAT v2.0 [83] to generate codon-based nucleotide and protein alignments, with parameters set to -m clustalw2 -f axt -k. The -k flag enabled integration with KaKs_Calculator v2.0 [84], applying the Yang–Nielsen (YN) model for Ka/Ks estimation. To avoid redundancy and ensure accurate comparisons, we retained only the longest transcript isoform from each genome or haplotype within a given group to represent that gene. We developed dedicated Python scripts to parse and filter the Ka/Ks output files, retaining only valid gene pairs with both Ka and Ks values, and excluding those with Ks equal to zero or showing anomalous values.

### Annotation and expression analysis of lincRNAs

We integrated both short-read RNA-Seq and long-read Iso-Seq transcriptomic data to annotate lincRNAs. Short-read RNA-Seq reads were aligned to the reference genome using Hisat2 v2.2.1 [56], and transcript assemblies were generated with StringTie v2.2.1 [61]. In parallel, long-read Iso-Seq data were aligned using minimap2 v2.28-r1209 [29] with the -L parameter to ensure accurate spliced alignment. The two transcript sets were then merged and filtered using FEELnc v0.2.1 [85], which removed transcripts shorter than 200 bp and those overlapping with annotated protein-coding genes. The remaining candidate transcripts were further evaluated for coding potential using two plant-specific deep learning-based tools: LncFinder-plant [34] and CPAT-plant [34]. In addition, we used diamond v2.0.15 [66] to align transcripts against the UniProt protein database [86] with an E-value cutoff of 1e-5. Only transcripts predicted as non-coding by all three approaches were retained as high confidence lncRNAs. Subsequently, lncRNAs were classified based on their genomic locations, and those located in intergenic regions without overlap with annotated protein-coding genes were designated as lincRNAs.

After lincRNA annotation, we first calculated the GC content, sequence length, and average exon length of both lincRNAs and mRNAs using tools such as Seqkit v0.8.1 [87]. Subsequently, featureCounts v2.0.6 [88] was used to quantify the raw read counts of lincRNAs and mRNAs across seven tissues: leaf, peel, pistil, pulp, seed, stamen, stem and leaf under environmental stress conditions. TPM values were then computed to estimate their expression levels. To assess tissue-specific expression, TPM values across the normal tissues were normalized. A transcript (lincRNA or mRNA) was considered tissue-specific if the ratio of its highest to lowest normalized TPM exceeded 3. The tissue with the highest normalized TPM was defined as the tissue of specific expression. Differentially expressed lincRNAs in response to environmental stress were identified using R package DESeq2 [89].

## Code availability

The HapGene pipeline is freely available on GitHub: https://github.com/JiaoLab2021/HapGene.

## Supplementary Material

Web_Material_uhag048

## Data Availability

The genome assemblies, and PacBio HiFi long reads of pummelo have been deposited into National Genomics Data Center (NGDC) (BioProject: PRJCA035310) and China National GeneBank DataBase (BioProject: CNP0006728). All transcriptome sequencing data generated in this study have been deposited in the NGDC under the accession number PRJCA042681. Lists of public RNA-Seq data used in this study are included in Table S11. Gene annotation files (GFF3) and genome assemblies for the three *Citrus* genomes analyzed in this study are deposited in the Figshare database (https://doi.org/10.6084/m9.figshare.30016324.v3).
